# Timed consumption of a New Zealand blackcurrant juice support positive affective responses during a self-motivated moderate walking exercise in healthy sedentary adults

**DOI:** 10.1186/s12970-019-0300-0

**Published:** 2019-08-02

**Authors:** Dominic Lomiwes, Birgit Ha, Nayer Ngametua, Natalie S. Burr, Janine M. Cooney, Tania M. Trower, Greg Sawyer, Duncan Hedderley, Roger D. Hurst, Suzanne M. Hurst

**Affiliations:** 1 The New Zealand Institute for Plant & Food Research Ltd. New Zealand Ltd, Private Bag, Palmerston North, 11030 New Zealand; 2 The New Zealand Institute for Plant & Food Research Ltd. MARC, Auckland, New Zealand Ltd, Private Bag 92169, Auckland, 1142 New Zealand; 3 The New Zealand Institute for Plant & Food Research Ltd., Private Bag 3230, Waikato Mail Centre, Hamilton, 3240 New Zealand

**Keywords:** Self-motivated exercise, Timed efficacy, Blackcurrant polyphenols, Affective response, Monoamine oxidase-B activity

## Abstract

**Background:**

Affective responses experienced during exercise are a significant determinant on exercise adherence. We have previously demonstrated that consumption of New Zealand (NZ) blackcurrants preserves cognition by attenuating the feeling of fatigue. This positive affective response correlated with the ability of blackcurrant polyphenols to support monoamine neurotransmission via inhibition of monoamine oxidase-B (MAO-B) activity. Here we explore how the consumption of a NZ blackcurrant juice (BJ) influenced affective responses and potential ergogenic action on the motivation to adhere to a low impact walking exercise.

**Methods:**

In a parallel randomized controlled study (Trial registration #: ACTRN12617000319370p, registered 28th February 2017, http://www.anzctr.org.au/), 40 healthy sedentary male and female participants drank a BJ or matched placebo (PLA) (*n* = 20 per group), 1 h prior to a self-motivated treadmill walk, where heart rate and affective responses (exertion [ES] or feeling / mood [FS]) scores) were recorded at 3 or 5 min intervals. Blood glucose, lactate, malondialdehyde (MDA) and platelet MAO-B activity were measured pre- and post-exercise and comparisons were conducted using with Student’s *t*-tests. Subjective data were analysed using 2-way ANOVA with appropriate post hoc tests.

**Results:**

Consuming a BJ 1 h prior to exercise caused a 90% decline in platelet MAO-B activity. The exercise had no significant (*p* > 0.05) effect on blood lactate, glucose or plasma MDA levels. Assessment of affective responses over the first 60 mins (adjusting for participant drop-out) revealed a time-dependent ES increase in both groups, with ES reported by participants in the BJ group consistently lower than those in the PLA group (*p* < 0.05). FS declined in PLA and BJ groups over 60 mins, but an inverse relationship with ES was only observed within the PLA group (r^2^ = 0.99, *p* = 0.001). Whilst the average time walked by participants in the BJ group was 11 mins longer than the PLA group (*p* = 0.3), and 30% of the BJ group achieving > 10 km compared to only 10% for the PLA group (*p* = 0.28), statistical significance was not achieved.

**Conclusion:**

Our findings demonstrate that drinking a polyphenolic-rich NZ blackcurrant juice 1 h prior to exercise supports positive affective responses during a self-motivated exercise.

## Background

Regular and appropriate exercise is associated with the prevention of chronic health problems such as heart disease and Type 2 diabetes [1–3] and with an increase in physical and mental functional abilities [4, 5]. To attain these protective and beneficial health effects, exercising daily for at least 30 min (mins) at a moderate intensity is recommended [3]. However, despite concerted efforts campaigning for the inclusion of daily exercise approximately 50% of adults fail to achieve these prescribed guidelines for exercise [3]. Also of concern is that 60% of individuals who commit to starting an exercise program drop-out within the first 6 months. Therefore, the challenge for those promoting physical activity extends beyond the encouragement of individuals to exercise through to ensuring that these committed individuals stay self-motivated when incorporating daily exercise as part of their habitual routine. Identifying foods or dietary supplements that specifically support the desire to exercise daily will enable an individual to adhere to an exercise program and maintain an active and healthy lifestyle.

A significant determinant on whether an individual will regularly participate in an exercise activity is their motivation to the exercise, i.e. their emotions and mood, which will include both positive and negative aspects. Muscle fatigue (defined as a reduction in maximal force generating ability [6]) experienced during exercise can be a major influence on an individual’s self-motivation to continue with the exercise and is dependent upon external influences such as the type of exercise, intensity and duration, environmental conditions (e.g. temperature) as well as the physical fitness and health status of the individual. Fatigue can by caused by both central and peripheral components, and whilst peripheral fatigue is a result of changes at or distal to the neuromuscular junction, central fatigue is due to a decrease in maximal voluntary activation [6]. The interplay and impact of both peripheral and central fatigue on motivation at different exercise intensities [7, 8] has led to the ‘dual-mode theory’ proposed by Ekkakakis et al. [9]. In this model, they postulate that changes in affective responses while exercising at low and moderate intensities are predominantly influenced by central factors. Whereas, the decline in affective responses during high intensity exercise is likely due to peripheral fatigue brought about by the physiological (i.e. neuromuscular) demand of the exercise rather than central fatigue. Furthermore, the mechanisms underpinning central responses to exercise are presently unclear, but appear to involve modulation of intrinsic brain factors. Alterations in brain neurochemistry has been proposed to have a significant role in mediating intrinsic motivation during prolonged exercise [10–13]. The monoamines serotonin, dopamine and noradrenaline have garnered the most attention with regards to exercise fatigue and motivation due to animal studies demonstrating the exercise-induced modulation of these neurotransmitters and their metabolites in localised areas of the brain [11, 12].

Dietary supplementation rich in polyphenolic compounds have been shown to support cognitive performance and mood via the modulation of monoamine nerve pathways in healthy adults and in those suffering with a cognitive disorder [14, 15]. In recent human nutrition intervention studies conducted by our group [16, 17], consumption of a New Zealand blackcurrant juice attenuated the decline in cognitive performance and reduced affective fatigue in healthy volunteers following a battery of cognitive tasks [16]. The preservation in cognitive performance was speculated to be mediated by the ability of blackcurrant-derived polyphenolic compounds to modulate monoamine neurotransmitters via the inhibition of monoamine oxidase-B (MAO-B) [17].

In this current study, we extend upon this current knowledge to explore the efficacy of timed consumption of a polyphenolic-rich juice made from New Zealand blackcurrants (delivering 4.8 mg total polyphenols/kg bodyweight) on the affective response in healthy sedentary individuals while they perform a self-motivated low impact walk on a treadmill designed not to induce peripheral fatigue or pain. The findings from this study contribute to the hypothesis that consumption of New Zealand blackcurrant polyphenolics facilitate positive affective responses to support exercise motivation and maintenance of a healthy active lifestyle.

## Materials and methods

### Subject selection

Forty male (*n* = 15) and female (*n* = 25) healthy volunteers between 20 and 59 years old were recruited from Massey University (Palmerston North campus), The New Zealand Institute for Plant & Food Research Ltd., and surrounding Palmerston North community. All volunteers were asked to omit foods, beverages and supplements high in antioxidants and polyphenols from their diet 24 h before the start of the study. Participants were also asked to refrain from any form of exercise 48 h prior to their exercise trial day. Individuals recruited for this study were healthy, but primarily sedentary, and selected for similar fitness characteristics, assessed by Baȇcke questionnaire [18], predicted VO_2_max values [19]. Participants also completed a health screening questionnaire to exclude those who were physically at risk from the exercise used in this study. Subjects were excluded from the study if they had known fruit (especially berry) allergies, blood-borne diseases (e.g. hepatitis), viral or bacterial illness, were taking medicines that affected blood properties (e.g. clotting), were pregnant or planning to get pregnant. Individuals participating in the study were excluded if they were unable to walk confidently at a moderate speed on a treadmill, carried current injury or recovering from injury received within the last 3 months, or exhibited chronic breathing and heart problems. In addition, since all participants recruited for this study were healthy and satisfied the study inclusion / exclusion criteria, consultation and approval from a health practitioner was not required and therefore sought at the discretion of the participant.

### Nutritional intervention

The New Zealand blackcurrant juice (BJ) was prepared from a blackcurrant juice concentrate made from New Zealand blackcurrants and kindly provided by The New Zealand Blackcurrant Co-operative Ltd. (Nelson, New Zealand). Polyphenol content was analysed by liquid chromatography-mass spectrometry (LC-MS) using a modified method described previously by Schrage and colleagues [20]. The amount and identification of blackcurrant polyphenolics was achieved by a combination of UV-visible and mass spectra against known compounds and calculated as μg/mL (Table 1). Total blackcurrant polyphenol concentration of 4.8 mg/kg bodyweight was given to participants. There is currently no recommended daily amount (RDA) for dietary plant-based polyphenolic compounds [21]. Therefore the total amount of blackcurrant polyphenolics used in this current study was selected using the bioavailability and bioactivity data reported in human nutrition intervention studies [22–25], including the bioefficacy of blackcurrant polyphenols on cognitive function [16, 17] and recovery from exercise-induced oxidative stress [22, 25]. The BJ concentrate was diluted in an opaque drink container with distilled water to a final volume of 200 mL. The 200 mL placebo (PLA) drink was prepared to contain the equivalent average amounts of glucose, fructose and vitamin C (9.2, 15.9 and 1.82 mg/kg bodyweight, respectively) present in the diluted BJ. In addition, drinks were freshly (within 30 mins) prepared on the morning of each participant’s trial day, with both the BJ and PLA drinks supplemented (350 μL) with blackcurrant flavouring (NI #12220, Formula Foods Corporation Ltd., Christchurch, New Zealand) to ensure that drinks exhibited a blackcurrant taste, minimizing the participant’s ability to distinguish between BJ and PLA drinks. Furthermore, the pre-exercise timing of drinking BJ (and PLA) adopted in this study was based upon the bioactivity results from Watson et al [16, 17], who showed that blackcurrant phenolic compounds were able to inhibit MAO-B activity and support positive affective responses within 1 h of consumption.

**Table 1:** Polyphenolic content of New Zealand blackcurrant juice

Polyphenol compound	BJ (μg/mL)
Anthocyanin	
Delphinidin 3-O-glucoside	1286
Delphinidin 3-O-rutinoside	6173
Cyanidin 3-O-glucoside	626
Cyanidin 3-O-rutinoside	7260
Petunidin 3-O-rutinoside	18
Pelargonidin 3-O-rutinoside	49
Peonidin 3-O-rutinoside	75
Phenolic acids	
Caffeic acid	15
Caffeoylhexose	49
*p*-Coumaroylhexose	56
*p*-Coumaric acid derivative	77
3-Caffeoylquinic acid	94
3-*p*-Coumaroylquinic acid	211
Ferulic acid glucoside	31
Flavan-3-ols	
Catechin	5
Epicatechin	4
Flavonols	
Myricetin 3-*O*-rutinoside	299
Myricetin 3-*O*-glucoside	164
Myricetin-malonylglucoside	35
Quercetin 3-*O*-rutinoside	194
Quercetin 3-*O*-glucoside	101
Quercetin malonylglucoside	14
Kaempferol 3-*O*-rutinoside	36
Kaempferol 3-*O*-glucoside	26
Aereusidin glucoside	15
Apigenin-hexoside	2
Myricetin	36
Quercetin	9
Total polyphenols	16,998

The polyphenolic composition of NZ blackcurrant juice (BJ) was measured by high pressure liquid chromatography (HPLC) against commercial polyphenolic standards and retention times. Results are shown as μg/mL of juice

### Trial format

#### (i) Pre-trial session

Participants underwent submaximal walking exercise tests on a treadmill ergometer (Motus M995TL, Queensland, Australia), using the methodology described by Ebelling et al. [19] to determine a moderate walking pace that didn’t evoke peripheral fatigue. Briefly, participants were initially asked to perform a 4 min walking exercise set at a 0% incline at a speed that brought their heart rate (HR) to between 50 and 70% of their age-predicted maximal HR. The treadmill incline was then increased to 5%, and participants were asked to maintain their walking to another 4 min to achieve a steady state HR (HRss). Next, using the predictive regression equation provided by Ebelling and colleagues, participants estimated VO_2_max (using their treadmill walking speed at HRss, age and sex) was calculated. Using this approach allowed us to normalise and select a treadmill walking pace according to each participants’ fitness levels that was predicted to minimize exercise-induced oxidative stress and neuromuscular (i.e. peripheral) fatigue (i.e. 80% of their predictive VO_2_max). In addition, participants were familiarised to the subjective visual analogue scales (VAS) that they would be required to respond to while exercising on their main trial day.

#### (ii) Main trial

This study employed a parallel, placebo-controlled, double-blinded design, with neither the study investigators nor the participants knowing what nutritional intervention was given or received. The random assignment of participants to a particular intervention groups and the preparation of BJ and PLA drinks were carried out by independent individuals who were not directly involved in the study. Participants were supplied with a list of foods, beverages and supplements high in antioxidants and polyphenols and instructed to omit these from their diet 24 h prior to their main trial day. The study was designed so all participants (irrespective of their trial day) performed the trial in the morning ~ 8 am. Participants were given a standardised meal bar (One Square Meal®, Cookie Time Ltd., Christchurch, New Zealand) to consume for breakfast at least 1 h prior to starting the trial. Upon arriving, participants completed an abbreviated Profile of Mood State (POMS) questionnaire [26]. Total mood disturbance was calculated by summing the scores for the negative subscales (tension, depression, anger, fatigue and confusion) and subtracting with the sum of the scores for the positive subscales (vigor and esteem-related effects). After donating a blood sample and fitted with a heart monitor (model AXN700 Polar Electro, Auckland, New Zealand), participants consumed either the BJ or PLA drink and relaxed for 1 h in the waiting area of the clinical facility and instructed to refrain from any form of moderate to strenuous physical exercise. During this time, participants spent the hour seated and engaged in sedentary activities such as reading, writing or watching (i.e. non-emotive) videos. Participants then donated another blood sample, taken to the exercise room and simply asked to walk for as long as they could, at their pre-determined personalized walking pace. They were not given a specific time or distance targets to achieve. To minimise their perception of walking time, participants exercised alone with all indicators of time (i.e. clocks, computer screens and personal watches, smart phones) removed. Participants were shown visual analogue scales (VAS) by the trial coordinator and asked to point to a number on a chart the presented their perceived (i) exertion (ES; 1 to 20) and (ii) mood/feeling (FS; − 5 to 5) at the time. VAS recordings were taken at the beginning of the exercise and then at 3 and 5 min intervals, which were randomly distributed to conceal the length of time that they have been exercising. In addition, no music or any form of encouragement (verbal or visual cues) was given and the participants were asked not to talk to trial coordinators unless they wanted to stop. The exercise was terminated after participants (i) completed walking for 2 h (end of trial), (ii) asked to stop or (iii) gave a FS below 0 for three consecutive time points, and the total duration (time and distance) of exercise was recorded. Aside from the trial coordinator, no other person was allowed in the room while the participant was exercising. A final blood sample was taken upon completion of exercise.

### Blood sampling

Whole blood (from finger pricks) was used to measure glucose and lactate with a ‘point of testing’ biosensors; Glucose (HemoCue® Glucose 201 DM System; Radiometer Pacific Ltd., Auckland, New Zealand), Lactate (Arkray Lactate Pro™ 2, Baden, Swizerland). Venous blood samples were collected into EDTA vacutainer tubes and immediately centrifuged at 600 *g*, 18 °C, for 5 mins to generate a platelet-rich plasma (PRP), which was further centrifuged at 2250 *g*, 18 °C, for 10 mins to generate a platelet pellet. Platelets were prepared using a modified method described by Watson et al [27] and frozen as a pellet at -80 °C until measurement of MAO-B activity. In addition, ~ 1 mL of the PRP was centrifuged at 300 *g*, 18 °C, for 10 mins and the cell-free plasma frozen at -80 °C until measurement of malondialdehyde (MDA).

### Platelet MAO-B activity

The MAO-B activity of platelet extracts was determined using an Amplex® Red Monoamine-B assay kit (Invitrogen, Thermo Fisher Scientific Ltd., Auckland) according to the manufacturer instructions. Briefly, platelet lysates were incubated with 0.05 mM clorgyline for 30 min at RT. Amplex Red substrate was then added to the platelet extract, plus H_2_O_2_ standards (0.01–2 mM) and phosphate buffer control. A change in fluorescence (FI) was measured at 37 °C over 10 mins (530–560 and 590 nm excitation and emission wavelengths, respectively) in a FLUOstar Omega plate reader (BMG FluoStar Optima, Alphatech Systems, Auckland, New Zealand). Platelet MAO-B activity was calculated, against H_2_O_2_ standards, and expressed as nM H_2_O_2_ produced /μg protein/min. All platelet extracts were assayed for MAO-B activity in duplicate with the coefficient of variation (CV) of replicate measures being < 10%.

### Plasma MDA levels (lipid peroxidation biomarker)

Plasma MDA were assessed by High Performance Liquid Chromatography (HPLC) using a modified method reported by Karatepe [28] against MDA standards. Briefly, MDA standards and plasma samples were precipitated with 5% (v/v) perchloric acid and the supernatant measured using a Shimadzu 20-series (Shimadzu Corporation, Kyoto, Japan) HPLC instrument equipped with a diode array detector. Calibration standards and samples were resolved using a Synergi™ 4 μM Polar-RP 80 Å column (Phenomenex®, Auckland, New Zealand) with a 95:5 (v/v) 30 mM monobasic potassium phosphate buffer (pH 3.6)-methanol mobile phase. The peak area and retention times of MDA in the standards and samples at 250 nm were evaluated using the Shimadzu LC solution software (Shimadzu Scientific Instruments, Auckland, New Zealand). MDA levels were calculated, against MDA standards and presented as μmol/L. All plasma samples were assayed in triplicate with the CV of replicate measures at < 10%.

### Statistical analysis

Data were analysed using Minitab® (version 18.1) and results expressed as means ± SEM for up to *n* = 20 participants in each intervention group. Two-sample Student’s *t-*tests were used to assess significant differences between the two intervention groups (placebo and blackcurrant) for each of the physiological and subjective variables measured. A repeated measures ANOVA was used to compare heart rate and subjective perceived ES and FS scores between the two intervention groups over time and determine significance for treatment effect. In addition, where participants stopped walking, their last recorded value was used for subsequent time points (a ‘last number carried forward’ approach [29]). Subsequent post hoc (least significant differences) tests were conducted on ES and FS scores following ANOVA analysis. In addition, Pearson correlations of FS and ES scores in each PLA and BJ intervention groups were conducted. Paired student *t*-tests were used to assess the effect of exercise on blood lactate, glucose and plasma MAO-B activity between baseline, pre-exercise and post-exercise time-points within the PL and BJ juice groups. Analysis to detect differences between PL and BJ groups within each time-point was conducted using unpaired t-tests. Statistical significance for all parameters was set at *p* < 0.05. In addition, the size effect between PLA and BJ groups was calculated using Cohen’s *d* index using the difference between the two group’s means divided by the average of their standard deviation. Subject power analysis with power 0.8 and a significance of *p* = 0.05 was calculated using Genstat STTEST procedure.

## Results

### Intervention

All participants completed the study and there were no reported adverse health effects from the BJ or PLA interventions.

### Pre-trial subject assessment

#### (a) Physical characteristics

Participants selected for this study displayed similar height & weight, were normally sedentary, and did not take part in any form of regular exercise (Table 2). Evaluation of their habitual activity using a Baȇcke questionnaire [18] revealed that both the work and sport index scores were similarly low in both the PLA and BJ groups. Assessment of their physical fitness in a pre-trial treadmill exercise session found that participants assigned to either the PLA or BJ groups exhibited similar (*p* > 0.05) (i) fitness profiles (Baȇcke questionnaire), (ii) predicted VO_2_max and HRmax scores (using Ebelling et al predictive regression equation [19]) and (iii) treadmill walking speed (~ 5.5 km/h).

**Table 2:** Participant physical and fitness evaluations

Variable	Mean ± SEM		*p* value
	PLA (*n* = 20)	BJ (*n* = 20)	
Sex			
Male	8	7	
Female	12	13	
Age (years)	29.9 ± 1.5	32.5 ± 2.4	0.370
Height (cm)	167.0 ± 2.2	167.9 ± 2.5	0.800
Weight (kg)	70.5 ± 3.3	69.0 ± 0.742	0.742
Predicted VO_2max_ (mL.kg^−1^.min^−1^)	45.54 ± 1.9	43.57 ± 1.4	0.417
Predicted HR_max_ (bpm)	190.0 ± 1.4	187.3 ± 2.4	0.332
Treadmill walking speed (km/h)	5.45 ± 0.10	5.50 ± 0.07	0.651
Habitual activity			
Work index	2.47 ± 0.16	2.35 ± 0.16	0.589
Sport index	2.63 ± 0.22	2.35 ± 0.23	0.377

Physical characteristics and habitual activity scores of participants randomly placed in either the placebo (PLA) or blackcurrant juice (BJ) intervention groups were measured using anthropometric measures and questionnaires. Results are mean ± SEM, n = 20 individuals per group, p value represents statistical difference from placebo group using Student’s 2-sample *t*-tests

#### (b) Mood evaluation

The POMS questionnaire is a recognized subjective tool to evaluate global mood changes in relation to varying exercise intensities in diverse populations (see review by Berger & Motl [30]). As a person’s mood prior to exercise may influence their affective state during exercise [31], a POMS questionnaire was used to determine the mood profile of study participants (Table 3). Completion of the POMS questions by participants, irrespective of nutrition intervention, immediately prior to the main trial found similar responses to the mood descriptors (*p* > 0.05) in all seven parameters; anger, confusion, depression, fatigue, tension, vigor and esteem related effect. When the overall total mood disturbance were calculated, the final scores did not significantly differ between individuals from the PLA and BJ groups (Table 3).

**Table 3:** Profile of mood states (POMS) of participants

Variable	Mean ± SEM		*P*-value
	PLA (*n* = 20)	BJ (*n* = 20)	
POMS variable			
Anger	0.74 ± 0.31	0.32 ± 0.17	0.250
Confusion	2.74 ± 0.78	2.00 ± 0.44	0.415
Depression	0.42 ± 0.18	0.26 ± 0.17	0.521
Fatigue	3.37 ± 0.62	2.32 ± 0.50	0.195
Tension	1.79 ± 0.38	1.37 ± 0.34	0.411
Vigour	7.0 ± 1.0	8.4 ± 1.1	0.371
Esteem related effects	11.7 ± 0.4	12.7 ± 0.7	0.216
Total mood disturbance	90.4 ± 2.2	85.2 ± 2.3	0.114

The emotional state of participants, randomly placed in either the placebo (PLA) or blackcurrant juice (BJ) intervention groups, was assessed using a POMS questionnaire. Results are mean ± SEM, n = 20 individuals per group, *p* value represents statistical difference from placebo group using Student’s 2-sample *t*-tests

### Platelet monoamine oxidase-B (MAO-B) activity

**Fig. 1:**
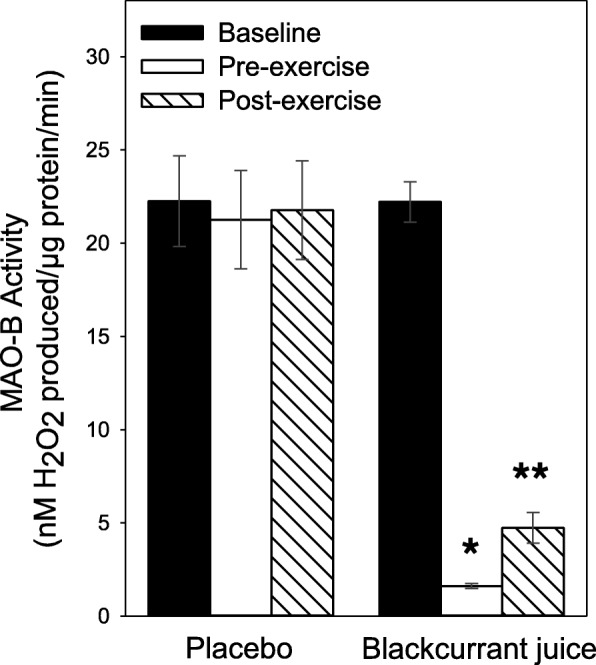
Timed New Zealand blackcurrant juice (BJ) consumption inhibits platelet monoamine oxidase-B (MAO-B) activity. Platelet MAO-B activity was measured in participants who had consumed either a placebo (PLA) or BJ drink 1 h prior to performing a low impact walking exercise. Platelets from blood collected at baseline (filled bars), pre- (unfilled bars) and post- (hatched bars) exercise were measured for MAO-B activity using commercial Amplex® Red Monoamine-B assay kit. Results are expressed as mean ± SEM, *n* = 20 individuals per group. ^*^p < 0.05 and **p < 0.01 represents statistical difference from baseline and pre-exercise values respectively within the PLA or BJ groups

A 90% (*p* < 0.001) reduction in platelet MAO-B activity was observed 1 h after the consumption of BJ; 22.1 ± 1.1 vs. 1.6 ± 0.1 nM H_2_O_2_ production/μg protein/min (Fig. 1). Lower levels of platelet MAO-B activity in the BJ group was still evident (*P* < 0.001) when participants had completed the walking exercise, even in those who walked for 2 h (4.7 ± 0.8 nM H_2_O_2_ production/μg protein/min). In contrast, consumption of the PLA, 1 h prior to exercise had no significant (*p* > 0.05) influence on platelet MAO-B activity: 22.2 ± 2.4 vs. 21.6 ± 2.6 vs. 21.7 ± 2.6 nM H_2_O_2_ production/μg protein/min, basal vs. pre- or post-exercise values. Furthermore, no significant difference in the baseline platelet MAO-B activity values was observed between PLA and BJ groups (*p* = 0.49).

### Blood glucose and lactate

**Fig. 2:**
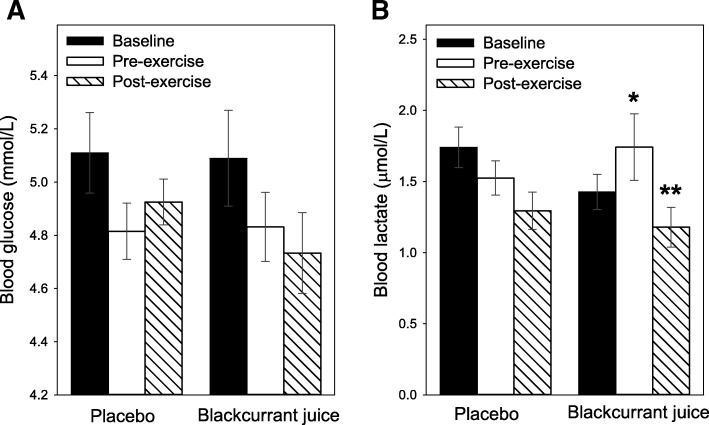
Timed consumption of New Zealand blackcurrant juice (BJ) modulates blood lactate but not glucose levels***.*** Blood glucose [**a**] and lactate [**b**] levels was measured in participants who had consumed either a placebo [PLA] or BJ drink 1 h prior to performing a low impact walking exercise. Blood glucose or lactate collected at baseline (filled bars), pre- (unfilled bars) and post- (hatched bars) exercise was measured using either HemoCue® Glucose 201 or Arkray Lactate Pro™ 2 biosensors, respectively. Results are expressed as mean ± SEM, n = 20 individuals per group. ^*^p < 0.05 and **p < 0.01 represents statistical difference from baseline and pre-exercise values respectively within each group

Consumption of either PLA (5.11 ± 0.2 vs. 4.8 ± 0.1 mmol/L, baseline vs. pre-exercise) or BJ (5.1 ± 0.2 vs. 4.8 ± 0.1 mmol/L, baseline vs pre-exercise) drinks had no effect on blood glucose levels (Fig. 2a). Furthermore, the walking exercise performed by study participants, had no impact on blood glucose levels, irrespective of distance walked or intervention (4.9 ± 0.1 or 4.7 ± 0.2 mmol/L, PLA or BJ post-exercise values). In terms of blood lactate, drinking the BJ 1 h prior to exercise caused a minor, yet significant (*p* = 0.048) 17% increase in pre-exercise blood lactate concentrations (1.4 ± 0.1 vs. 1.7 ± 0.2 μmol/L, baseline vs. pre-exercise levels (Fig. 2b). This transient increase in blood lactate declined during the treadmill walk revealing a post-exercise blood lactate level 30% lower (*p* = 0.02*)* than pre-exercise levels. No increase in baseline blood lactate was observed 1 h after the consumption of the PLA drink (1.7 ± 0.1 vs. 1.5 ± 0.2 μmol/L, baseline vs. pre-exercise values, *p* = 0.08), and although lactate levels were 15% lower in post-exercise blood it was not statistically (*p* = 0.09) different from pre-exercise values (Fig. 2b).

### Exercise-induced peripheral changes

**Fig. 3:**
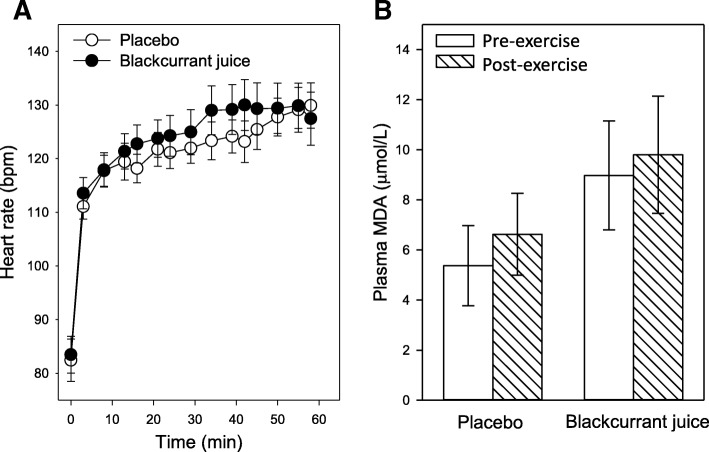
Timed consumption of New Zealand blackcurrant juice (BJ) has no impact on exercise-induced changes in heart rate (HR) or oxidative stress biomarker malondialdehyde (MDA). Participants consumed either a placebo (PLA) (open circles) or BJ (filled circles) drink 1 h prior to being performing a low impact treadmill waking exercise. **a** HR was measured using a PolarTM heart monitor and expressed as mean ± SEM beats per minute (bpm). **b** Plasma MDA was measured pre- (unfilled bars) and post- (hatched bars) exercise by high pressure liquid chromatography (HPLC). Results are expressed as mean ± SEM, *n* = 20 individuals per group

Participants randomly selected into the two intervention groups displayed similar pre-exercise HR (82 ± 4 vs. 83 ± 3 bpm, PLA vs. BJ). An initial time-dependent increase in HR was observed once the participants started to walk on the treadmill, which plateaued after 10 mins walking and then remained relatively constant over the rest of the exercise (Fig. 3a). Furthermore, participant’s post-exercise HR were similar in both intervention groups irrespective of when they finished walking (126 ± 1 vs.127 ± 2 bpm, PLA vs. BJ) and were below their predicted HRmax (Table 2). Assessment of exercise-induced oxidative stress showed no significant increase in plasma MDA levels in participants upon completion of the exercise trial (Fig. 3b). This was irrespective of the time walked, or intervention group: PLA; 5.4 ± 1.6 vs. 6.6 ± 1.6 μmol/L, *p* = 0.115 (pre- vs. post-exercise) or BJ; 8.2 ± 2.2 vs. 9.8 ± 2.3 μmol/L, *p* = 0.830 (pre-vs. post-exercise). Furthermore, pre-exercise plasma MDA in both BJ and PLA group were similar (*p* = 0.09).

### Walking compliance and distance

**Fig. 4:**
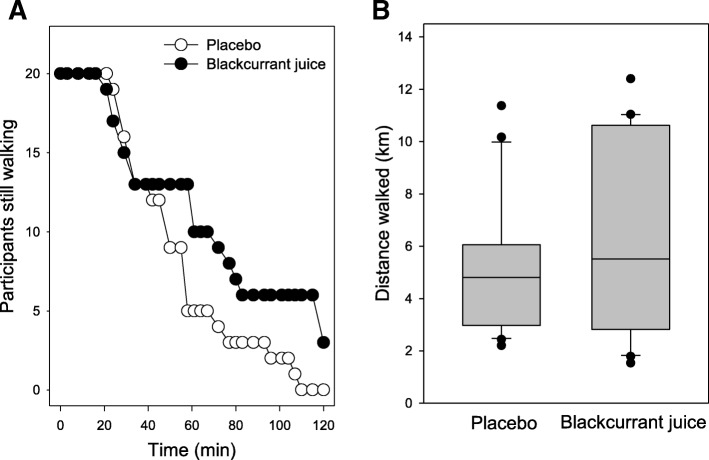
Timed consumption of New Zealand blackcurrant juice (BJ) has a marginal influence on the drop-out time and distance walked by participants*.* **a** Exercise drop-out time and [**b**] distance walked was recorded during a low impact treadmill walking exercise. Results are expressed as either [**a**] a step-wise plot of participant’s drop-out time in placebo (PLA, open circles) or BJ (filled circles) groups or [**b**] Box and Whisker plot of distance walked by participants in the two groups; n = 20 per group; centre line of box indicates median, box encloses the middle 50% of values; whiskers mark the 10 and 90% percentiles of the data, and dots mark observations beyond those

In this study, we found that none of the participants requested to stop once they had commenced the walking exercise, and with the exception of three participants in the BJ group who walked for 2 h, the exercise for other participants was terminated after they indicated a FS of zero or below for three consecutive time-points. The number of active participants declined as the exercise time progressed (Fig. 4a). After 60 mins, approximately half the number of participants were still walking; PLA (8/20) and BJ (11/20) groups. Beyond 60 mins, the number of participants still walking in the PLA group continued to rapidly decline and by 110 mins, all participants within this group had dropped out. In contrast, the drop-out rate in the BJ group was lower, with 15% still walking at 2 h. The average distance walked by participants in the intervention groups showed no overall significant differences (5.1 ± 0.6 vs. 6.2 ± 0.8 km, PLA vs. BJ, *p* = 0.28, Fig. 4b), with a small-medium effect size of *d* = 0.44 and a predicted subject power analysis of *n* = 67. The average time walked by participants within the RBJ group was 11 mins longer than within the PLA group (66.9 ± 8.5 vs. 55.9 ± 5.6 mins, RBJ vs. PLA). This was non-significant (*p* = 0.3) and displayed a small effect size of *d* = 0.35 with a predicted subject power analysis of *n* = 103. Closer examination of distance walked by participants revealed that 50% of participants in both the PLA and BJ walked a distance of 5 km or more (Fig. 4b). Only 10% of participants in the PLA group walked a distance greater than 10 km, however, compared with 30% of participants in the BJ group (*p* = 0.28).

### Affective responses

**Fig. 5:**
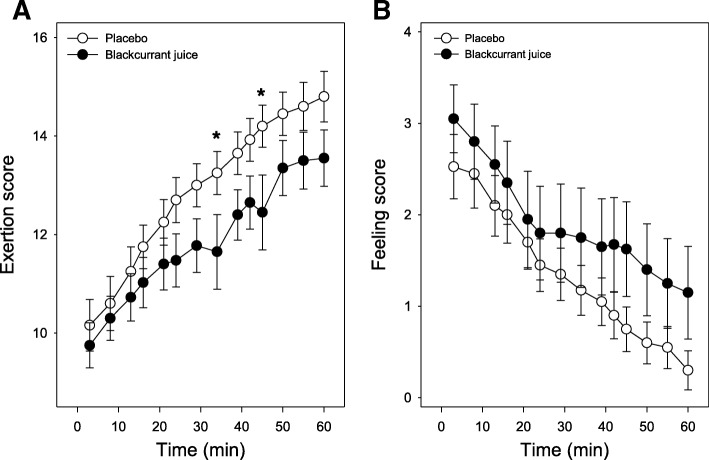
Timed consumption of blackcurrant juice (BJ) modulates affective responses during a moderate walking exercise. Affective responses for perceived exertion [**a**] and feeling/mood [**b**] by participants who had either consumed placebo (PLA, open circles) or BJ drink (filled circles) 1 h prior to performing a low impact exercise were assessed visual analogue scale [VAS] sheets at 3 or 5 mins intervals. Results are expressed as mean ± SEM, *n* = 20 individuals per group. *p < 0.05 represents statistical difference from PLA at corresponding walking time

Since the majority of participants dropped-out before the 2 h completion time (Fig. 4), we explored time-dependent changes in affective responses over the first 60 mins of the trial (Fig. 5). To account for participants dropping-out during this period, we used a ‘last number carried forward’ [29] approach to explore time-dependent changes in participants’ perceived ES and FS responses over this period.

#### (a) Perceived exertion response

Participants from both the PLA and BJ groups recorded a similar time-dependent increase in perceived exertion scores (ES) Fig. 5a; overall time effect in repeated-measures ANOVA *p* < 0.001. Beyond 20 mins of exercise, the BJ group showed a trend (*p* = 0.086) towards lower ES scores being reported (treatment effect in repeated measures ANOVA, between PLA (12.9 ± 0.4) and BJ (11.9 ± 0.5) so that ES scores in the BJ group were significantly (*p* < 0.05) lower at 34 and 45 mins from the onset of walking compared to that reported by participants in the PLA group. Although ES scores reported by participants at 60 mins were no longer significant (*p* = 0.06), the trend of a lower ES score was still evident (14.8 ± 0.58 vs. 13.5 ± 0.5, BJ vs. PLA group). Furthermore, calculation of effect sizes at 21, 40 and 60 mins revealed *d* = − 0.37, − 0.58 and − 0.5 respectively, with predicted subject power analyses of *n* = 89, 38 and 50 respectively.

#### (b) Perceived mood / feeling response

A time-dependent decrease in perceived mood / feeling scores (FS) was recorded by participants in both intervention groups over the first 20 mins of walking; 3.0 ± 0.4 to 1.9 ± 0.5 vs. 2.5 ± 0.3 to 1.7 ± 0.3, 0 to 20 mins in BJ vs. PLA group (Fig. 5b). This downward “feeling/mood” trend (recorded or predicted) continued in the PLA group over the next 40 mins, with majority the participants reporting a FS of 1 or 0 after 60 mins walking. FS recorded by participants in the BJ group remained relatively stable between 20 and 40 mins, and despite being higher than the FS observed in the PLA was not statistically different (*p* > 0.05) and showed minimal effect sizes of *d* = 0.13 and 0.29 at 21 and 40 mins, respectively, with predicted subject power analyses of *n* = 753 and 150 respectively. After 40 mins, the FS reported in the BJ groups showed a gradual decrease, with reported scores still higher than those recorded by the PLA group. Although not significant (p = 0.06), this trend continued up to 60 mins from the onset of walking, with the average FS reported by participants in the BJ group consistently higher than those recorded by the PLA group (1.15 ± 0.51 vs. 0.3 ± 0.21, BJ vs. PLA group). In addition, a medium effect size of *d* = 0.48 was observed at 60 mins (with predicted subject power analyses of *n* = 56), which was similar to medium effect size (*d* = 0.5) calculated for perceived fatigue at the same time.

### (c) Relationship between perceived exertion (ES) and mood/feeling (FS) responses

**Fig. 6:**
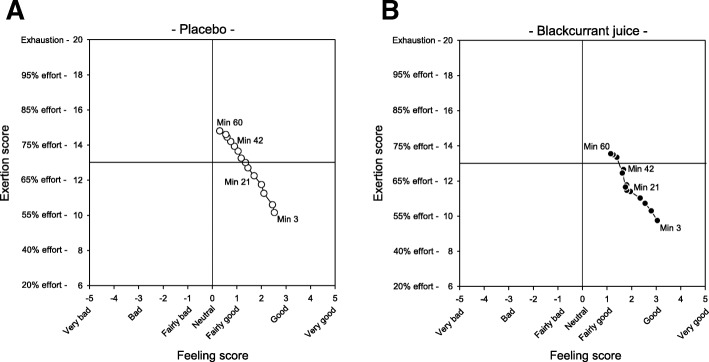
Timed consumption of New Zealand blackcurrant juice (BJ) skews the inverse relationship between perceived exertion (ES) and feeling/mood (ES) scores during exercise. Correlation between ES and FS (assessed using visual analogue scales [VAS]) reported by participants who had consumed either [**a**] placebo (PLA) or [**b**] BJ drinks 1 h prior to performing a low impact walking exercise is expressed as circumplex plots. Results are calculated as ‘last number carried forward’ and expressed as mean ± SEM for each intervention group

The relationship between ES and FS values (recorded or predicted) in the PLA group indicated a strong inverse linear relationship (r^2^ = 0.99, *p* = 0.001) over a 60 mins period of walking (Fig. 6a). A circumplex plot of ES and FS affective responses recorded by participants in the BJ group showed an initial inverse relationship between ES and FS over the first 21 mins of walking (Fig. 6b) similar to that observed in the PLA group. After this time the relationship between FS and ES became skewed, with participants recording static FS over the following 45 mins (Fig. 5b) despite a continued gradual increase in perceived ES.

## Discussion

In this preliminary study, timed consumption of a polyphenolic-rich juice made from New Zealand blackcurrants 1 h prior to exercise supports positive central affective responses during a self-motivated low impact walking exercise. Motivation and commitment to exercise is largely driven by an individual’s affective response. This is a complicated process involving a number of factors, including an individual’s psychological disposition, physical fitness, and importantly the exercise type, intensity, duration and environmental settings [32]. Previous studies by us [16, 17] revealed a time-dependent inhibition of MAO-B activity after the consumption of a polyphenolic-rich BJ and subsequent modulation of affective-regulating monoamine neurotransmitters. These findings suggest that acute bioavailability and bioactivity of blackcurrant polyphenolics may also support positive affective responses, like motivation, during exercise. Here we employ a parallel study design (minimize any exercise learning effect) to explore the capability of the BJ to assist positive affective responses (such as vigilance, mood, and motivation) in the context of adhering to a treadmill walking exercise for 2 h, personalized for participants fitness so as not to evoke peripheral fatigue.

To maximize the potential efficacy of the BJ, recruited individuals had similar physical characteristics and, by using a combination of questionnaires and a pre-trial exercise assessment, comparable physical fitness and mood profiles. Previous studies show that exercising at a high intensity above the lactate and ventilatory thresholds rapidly leads to neuromuscular (i.e. peripheral) fatigue and concomitant decline in exercising motivation. Here the exercise involved individuals walking on a treadmill at an intensity (calculated using the submaximal exercise intensity formula reported by Ebbeling et al [19]) predicted to minimise peripheral fatigue. Peripheral fatigue, however, was not measured in this study, and since there is a strong link between central and peripheral fatigue [6], we cannot exclude the possibility that changes in peripheral fatigue might contribute participant’s perceived exertion and drop-out rates, especially after 60 mins. Nevertheless, all participants found walking at the ~ 5.5 km/h pace easy to do and displayed similar exercise-induced steady-state heart rates with marginal, non-significant, changes in post-exercise blood lactate and an oxidative stress biomarker (MDA), irrespective of how long they walked for. Furthermore, support for this approach when exploring affective responses to exercise is shown by others [8, 33] who found that positive affective responses were observed in healthy, but untrained, individuals who performed an exercise below their VO_2_max independent of peripheral influences such as muscle fatigue and pain experienced in high impact exercise. In addition, both the BJ and PLA drink consumed by the participants in this study contained the same amount of sugar, which also may influence affective responses. Here we found no fluctuations in blood glucose after the consumption or either PLA or BJ, or during the walk on the treadmill. However, consumption of the BJ, without exercise, did cause a small increase in participants’ blood lactate levels, which was transient, although were lower than baseline levels by the end of the walking exercise. The mechanisms underlying these changes in the blood lactate profile are unclear as the participants relaxed prior to exercise and kept to the walking pace instructed by the trial coordinator during the treadmill exercise. Since lactate affect cognitive energy utilization [34], we cannot exclude the possibility that transient changes in blood lactate observed in the BJ group, prior to and during exercise, may be the result of blackcurrant-derived polyphenols potential influence on energy glycolytic metabolism and central affective responses.

Applying a low impact treadmill walking pace in this study that was predicted to minimise peripheral fatigue and exercise-induced oxidative stress enabled us to explore the efficacy of the BJ (rich in polyphenols) in supporting a positive affective response, and motivation to exercise. Participant affective responses varied over the length of the walk and were linked to participant walk time and drop-out rate. Participant’s perception of fatigue in the PLA group was consistently higher than those who consumed the BJ drink over the first 60 mins walking period. This was despite participants’ peripheral responses (HR, blood lactate and MDA) to the exercise being similar as well as displaying similar pre-exercise fitness scores. Affective mood responses decreased with walk time in both intervention groups, however after 20 mins differences between the PLA and BJ groups were observed. This, however, coincided with the beginning of participant’s drop-out and therefore, due to the small number of individuals taking part in this preliminary study, cannot exclude the possibility that the participants still walking after 60 mins may have exhibited an innate self-motivation to exercise irrespective of the nutrition group they were assigned to, although calculation of Cohen’s *d* index revealed a medium effect size indicating that an increase in participant numbers (>*n* = 50) may show significant treatment effect (*p* = 0.05) between PLA and BJ groups. Furthermore, to minimize (although not exclude) the influence of participant drop-out a ‘last number carried forward’ analysis, which is the recommended statistical approach used in long-term clinical intervention studies to account for patient drop-out [29] was applied. Affective responses by participants in the PLA group revealed a clear inverse linear relationship between perceived exertion and mood. This was not as apparent in the BJ group, suggesting that blackcurrant-derived polyphenolic compounds maybe having an impact on central affective responses resulting in the skewed inverse relationship observed between the perceived EF and FS during exercise. The cause for this, although unknown, may involve blackcurrant polyphenols modulating neural pathways primarily involved in perceived fatigue, and supports this observation reported by us [16] and others [35, 36].

In addition to individual physical fitness variations, motivation studies show that environmental settings whilst exercising are also important for exercise adherence. Listening to music [37–39] or being coached [40, 41] while exercising improves compliance and has a positive effect on mood. Here, participants conducted the treadmill walking exercise at the same time of day (i.e. ~ 8 am), in a room that was set-up to eliminate external factors (windows were obstructed, any time indicators or visuals cues on walls or furniture within participant’s sight were masked or removed and no one was allowed into the room except the trial coordinator to collect subjective data). Participants were, therefore, reliant upon self-motivation to complete the treadmill exercise, and also interaction between participant and trial coordinator taking the subjective measures were keep to a minimum, we cannot exclude that it may have had an impact as the walk time increase and participants began to get bored and the temptation to quit greater. Feedback voluntarily disclosed by participants (irrespective of nutrition intervention) at the conclusion of their exercise revealed that they were bored and most would have walked longer if they had been allowed to listen to music or had been coached. Participants within the PLA group, in particular, reported a higher degree of boredom and had a higher drop-out rate within the first 30 mins. This observation lends support to the hedonic principle of adhering to exercise (over-viewed by Williams [32]), whereby allowing individuals to have self-control over exercising conditions (i.e. intensity, pace and environmental settings) produces a sustainable positive affective response. Since individuals do not typically exercise in environments devoid of these external factors, further studies are required to determine whether the efficacy of BJ on central affective responses would be supported and/or enhanced by additional self-motivating factors (i.e. music) when adhering to regular exercise regimes.

Polyphenols (especially anthocyanins) are the predominant flavonoids present in berryfruit and the acute functional benefits attained from consuming berryfruit is dependent on their bioavailability and bioactivity. Human feeding studies [16, 26, 42–45] show a time-dependent increase in polyphenolic compounds and/or metabolites within the plasma 1 h after consumption of berryfruit, including blackcurrant [16, 26]. This is shown to coincide with acute biochemical and physiological changes including increased peripheral blood flow [46] and endothelial function [47] in healthy adults. In addition, regular consumption of berries has been associated with long-term cognitive health [48, 49] that may involve polyphenolic and/or metabolite liver transformation, tissue accumulation and/or colon microflora [43, 50, 51]. Here in this current study, we applied previous knowledge of the acute blackcurrant polyphenolic compounds bioavailability reported by us [17, 26] and others [42, 46] together with the temporal MAO inhibition profile after the consumption of a polyphenolic-rich BJ reported by Watson et al. [16, 17] to select a suitable BJ dose and pre-exercise consumption time to maximize the potential influence on positive affective responses during a low impact exercise. Indeed, we found that plasma collected 1 h after BJ (4.8 mg/kg bodyweight) consumption showed a dramatic acute decline (> 90%) in platelet MAO-B activity, which was still detectable in participant’s plasma once they had stopped exercise, even those who walked for 2 h. Furthermore, although the plasma polyphenolic bioavailability profile and identification of the polyphenol bioactive(s) was not the focus of this study, the observed post-consumption bioefficacy of BJ on MAO-B activity supports Watson’s et al. [16, 17] observations of an acute decline in MAO-B activity after the consumption of BJ. Since the decline in MAO-B activity correlates with the preservation of monoamine neurotransmission [52] and reduced perception of fatigue while conducting a set battery of cognitive tasks [16], it is possible that, in this current study, the inhibitory action of pre-exercise consumption of BJ on MAO-B activity detected in participants for the length of their exercise may have influenced and/or contributed to the overall positive affective response observed in this group.

MAO-A and MAO-B are both involved in the degradation of various monoamine neurotransmitters including dopamine, serotonin and norepinephrine. Although both isoenzymes are active in the central nervous system, only MAO-B is found in human blood platelets. The pharmacological inhibition of brain MAO-B activity has been used to treat those diagnosed with neurological diseases and depression [53], potentially through their neuroprotective properties in reducing the metabolism of monoamines. There is a good correlation between platelet and central nervous system MAO-B activity and changes in platelet MAO-B activity are shown to be a suitable biomarker for fluctuations in monoamine neurotransmitters and therefore the affective response [16, 17]. Moreover, there is some evidence that exercise influences monoamine activity. Platelet MAO-B activity was found to progressively increase following short successive cycling bouts at increasing intensities then declined once the exercise intensity reached 40% of an individual’s maximal tolerance in healthy male volunteers [54]. Further, MAO-B activity was found to be inversely correlated to subjective ratings of perceived exertion following a bout of maximal exercise [55]. These findings suggest the involvement of MAO-B in the metabolism of key neurotransmitters during exercise, thereby influencing affect and motivation, which may be more prominent during low and moderate exercise intensities when exercise-induced MAO-B activity is optimal. Here comparison between platelet MAO activity and the time walked by participants in the BJ group revealed a tentative (r^2^ = 0.17, *p* = 0.12) inverse relationship that was not evident in the PLA group. Whilst it can be speculated that the inhibitory action of blackcurrant polyphenols on MAO-B activity modulates central affective responses and motivation to exercise, the lower drop-out rate observed in the BJ group in this preliminary study was not significant.

## Conclusion

Findings from this preliminary study provides evidence that timed consumption of a polyphenolic-rich juice made from New Zealand blackcurrants 1 h prior to exercise supports positive affective responses during a low impact walking exercise in healthy sedentary adults. Future clinical studies extrapolating the link between blackcurrant-derived polyphenolic compounds, monoamine neurotransmission (via inhibition of MAO-B activity) and positive affective responses will enable the determination of potential ergogenic action for self-motived exercise adherence to be established.

## Data Availability

The data sets for this manuscript are not publicly available because of commercial sensitivity. Requests to access the datasets should be directed to the corresponding author.

## References

[1] Petersen AM, Pedersen BK (2005). The anti-inflammatory effect of exercise. J Appl Physiol.

[2] Knowler WC, Barrett-Connor E, Fowler SE (2002). Reduction in the incidence of type 2 diabetes with lifestyle intervention or metformin. New Eng J Med.

[3] Haskell WL, Lee IM, Pate RR (2007). Physical activity and public health: updated recommendation for adults from the american college of sports medicine and the american heart association. Circulation.

[4] Oguma Y, Sesso HD, Paffenbarger RS (2002). Physical activity and all cause mortality in women: a review of the evidence. Br J Sports Med.

[5] Herring MP, Puetz TW, O'Connor PJ (2012). Effect of exercise training on depressive symptoms among patients with a chronic illness: a systematic review and meta-analysis of randomized controlled trials. Arch Intern Med.

[6] Wan JJ, Qin Z, Wang P, Sun Y, Liu X (2017). Muscle fatigue:general understanding and treatment. Exp Mol Med.

[7] Frazão DT, de Farias Junior LF, Dantas TCB (2016). Feeling of pleasure to high-intensity interval exercise is dependent of the number of work bouts and physical activity status. PloS One.

[8] Parfitt G, Rose EA, Burgess WM (2006). The psychological and physiological responses of sedentary individuals to prescribed and preferred intensity exercise. Br J Health Psychol.

[9] Ekkekakis P, Hall EE, Petruzzello SJ (2008). The relationship between exercise intensity and affective responses demystified: to crack the 40-year-old nut, replace the 40-year-old nutcracker!. Ann Behav Med.

[10] Heijnen S, Hommel B, Kibele A, et al. Neuromodulation of aerobic exercise - a review. Front Psychol. 2016;6. 10.3389/fpsyg.2015.01890. 10.3389/fpsyg.2015.01890 PMC4703784 26779053

[11] Basso JC, Suzuki WA (2017). The effects of acute exercise on mood, cognition, neurophysiology, and neurochemical pathways: a review. Brain Plasticity.

[12] Cordeiro LMS, Rabelo PCR, Moraes MM, et al. Physical exercise-induced fatigue: the role of serotonergic and dopaminergic systems. Brazilian J Med Biol Res. 2017;50. 10.1590/1414-431x20176432. 10.1590/1414-431X20176432 PMC5649871 29069229

[13] Davis JM, Bailey SP (1997). Possible mechanisms of central nervous system fatigue during exercise. Med Sci Sports Exerc.

[14] Gomez-Pinilla F, Nguyen TTJ (2012). Natural mood foods: the actions of polyphenols against psychiatric and cognitive disorders. Nutr Neurosci.

[15] Haskell-Ramsay CF, Stuart RC, Okello EJ (2017). Cognitive and mood improvements following acute supplementation with purple grape juice in healthy young adults. Eur J Nutr.

[16] Watson A, Haskell-Ramsay CF (2015). Acute supplementation with blackcurrant extracts modulates cognitive functioning and inhibits monoamine oxidase-B in healthy young adults. J Func Foods.

[17] Watson A, Scheepens a, et al. The pharmacodynamic profile of “blackadder” blackcurrant juice effects upon the monoamine axis in humans: a randomised controlled trial. Nutr Neurosci. 2018:1–10. 10.1080/1028415X.2018.1525950. 10.1080/1028415X.2018.1525950 30289026

[18] Baecke JA, Burema J, Frijters JE (1982). A short questionnaire for the measurement of habitual physical activity in epidemiological studies. Am J Clin Nutr.

[19] Ebbeling CB, Ward A, Puleo EM (1991). Development of a single-stage submaximal treadmill walking test. Med Sci Sports Exerc.

[20] Schrage B, Stevenson D, Wells RW (2010). Evaluating the health benefits of fruits for physical fitness: a research platform. J Berry Res.

[21] Pojer E, Mattivi F, Johnson D (2013). The case for anthocyanin consumption to promote human health: a review. Com Rev Food Sci & Food Safety.

[22] Lyall KA, Hurst SM, Cooney J (2009). Short-term blackcurrant extract consumption modulates exercise-induced oxidative stress and lipopolysaccharide-stimulated inflammatory responses. Am J Physiol Regul Integr Comp Physiol.

[23] Willems ME, Myers SD, Gault ML (2015). Beneficial physiological effects with blackcurrant intake in endurance athletes. Int J Sport Nutr Exerc Metab.

[24] Cook Matthew David, Myers Stephen David, Gault Mandy Lucinda, Willems Mark Elisabeth Theodorus (2017). Blackcurrant Alters Physiological Responses and Femoral Artery Diameter during Sustained Isometric Contraction. Nutrients.

[25] Hurst RD, Lyall KA, Roberts JM, et al. Consumption of an anthocyanin-rich extract made from New Zealand blackcurrants prior to exercise may assist recovery from oxidative stress and maintain circulating neutrophil function. Front Nut. 2019;6. 10.3389/fnut.2019.00073. 10.3389/fnut.2019.00073 PMC6548855 31192216

[26] Grove JR, Prapavessis H (1992). Preliminary evidence for the reliability and validity of an abbreviated profile of mood states. International J Sport Psychol.

[27] Watson P, Hasegawa H (2005). Acute dopamine/noradrenaline reuptake inhibition enhances human exercise performance in warm, but not temperate conditions. J Appl Physiol.

[28] Karatepe M (2004). Simultaneous determination of ascorbic acid and free malondialdehyde in human serum by HPLC-UV. LCGC Asia Pacific.

[29] Guideline on missing data in confirmatory clinical trials, in European Medicine Agency Guideline (Committee for Medicinal Products for Human Use). 2011, European Medicines Agency: United Kingdom.

[30] Berger BG, Motl RW (2000). Exercise and mood: a selective review and synthesis of research employing the profile of mood states. J Appl Sport Psychol.

[31] Terry PC & Karageorghis CI: Psychological effects of music in sports and exercise: An update on theory research and application. In M. Katsikitis (ed.), Psychology bridging the Tasmen: Science, culture and practice - Proc Joint Conference of Aus & NZ Psychol Societies, 2006:415–419.

[32] Williams DM (2008). Exercise, affect, and adherence: an integrated model and a case for self-paced exercise. J Sport Exerc Psychol.

[33] Ekkekakis P, Hall EE, VanLanduyt LM (2000). Walking in (affective) circles: can short walks enhance affect?. J Behav Med.

[34] Schurr A, Gozal E (2011). Aerobic production and utilization of lactate satisfy increased energy demands upon neuronal activation in hippocampal slices and provide neuroprotection against oxidative stress. Front Pharmacol.

[35] Deley Gaëlle, Guillemet Damien, Allaert François-André, Babault Nicolas (2017). An Acute Dose of Specific Grape and Apple Polyphenols Improves Endurance Performance: A Randomized, Crossover, Double-Blind versus Placebo Controlled Study. Nutrients.

[36] Reed RA, Mitchell ES, Saunders C, O'Commor PJ (2019). Acute low and moderate doses of a caffeine-free polyphenol-rich coffeeberry extract improve feelings of alertness and fatigue resulting from performance of fatiguing cognitive tasks. J Cognitive Enhancement.

[37] Clark IN, Baker FA, Taylor NF (2016). The modulating effects of music listening on health-related exercise and physical activity in adults: a systematic review and narrative synthesis. Nordic J Music Therapy.

[38] Laukka P, Quick L (2013). Emotional and motivational uses of music in sports and exercise: a questionnaire study among athletes. Psychol of Music.

[39] Ramji R, Aasa U, Paulin J (2016). Musical information increases physical performance for synchronous but not asynchronous running. Psychol Music.

[40] Black SJ, Weiss MR (1992). The relationship among perceived coaching behaviors, perceptions of ability, and motivation in competitive age-group swimmers. J Sport Exerc Psychol.

[41] Sarrazin P, Vallerand R, Guillet E (2002). Motivation and dropout in female handballers: a 21-month prospective study. Eur J Soc Psychol.

[42] Alimbetov YJ, George T, Gordon MH, Lovegrove JA (2011). A randonised trial to investigate the effects of acute consumption of blackcurrant juice on markers of vascular reactivity and bioavailability of anthocyanins in humjan subjects. Eur J Clin Nutr.

[43] Zhong S, Sandhu A, Edirisinghe I, Burton-Freeman B (2017). Characterisation of wild blueberry polyphenols bioavailability and kinetic profile in plasma over 24-h period in human subjects. Mol Nutr Food Res.

[44] Schon C, Wacker R, Micka A (2018). Bioavailability study of Maqui berry extract in healthy subjects. Nutrients.

[45] Manach C, Williamson G, Morand C (2005). Bioavailability and bioefficacy of polyphenols in human. I. Review of 97 bioavailability studies. Am J Clin Nutr.

[46] Matsumoto H, Takenami E, Iwasaki-Kurashige K (2005). Effects of blackcurrant anthocyanin intake on peripheral muscle circulation during typing work in humans. Eur J Appl Physiol.

[47] Rodriguez-Mateos A, Rendeiro C, Bergillos-Meca T (2013). Intake and time dependence of blueberry flavonoid-induced improvements in vascular function: a randomized, controlled, double-blind, crossover intervention study with mechanistic insights into biological activity. Am J Clin Nutr.

[48] Lamport DJ, Lawton CL, Merat N (2016). Concord grape juice, cognitive function, and driving performance: a 12-wk, placebo-controlled, randomized crossover trial in mothers of preteen children. Am J Clin Nutr.

[49] Scholey AB, French SJ, Morris PJ (2010). Consumption of cocoa flavanols results in acute improvements in mood and cognitive performance during sustained mental effort. J Psychopharmacol.

[50] D'Archivio M, Filesi C, Vari R (2010). Bioavailability of polyphenols:status and controversies. Int J Mol Sci.

[51] Ozdal Tugba, Sela David A., Xiao Jianbo, Boyacioglu Dilek, Chen Fang, Capanoglu Esra (2016). The Reciprocal Interactions between Polyphenols and Gut Microbiota and Effects on Bioaccessibility. Nutrients.

[52] Bortolato M, Chen K, Shih JC (2008). Monoamine oxidase inactivation: from pathophysiology to therapeutics. Adv Drug Deliv Rev.

[53] Youdim Moussa B H, Bakhle Y S (2009). Monoamine oxidase: isoforms and inhibitors in Parkinson's disease and depressive illness. British Journal of Pharmacology.

[54] Gawel MJ, Glover V, Burkitt M (1981). The specific activity of platelet monoamine oxidase varies with platelet count during severe exercise and noradrenaline infusion. Psychopharmacology.

[55] Harro J, Oreland L (2016). The role of MAO in personality and drug use. Prog Neuro-Psychopharm Biol Psych.

